# Human *Lactobacillus* Strains from the Intestine can Suppress IgE-Mediated Degranulation of Rat Basophilic Leukaemia (RBL-2H3) Cells

**DOI:** 10.3390/microorganisms4040040

**Published:** 2016-10-27

**Authors:** Gaku Harata, Fang He, Kyoko Takahashi, Akira Hosono, Kenji Miyazawa, Kazutoyo Yoda, Masaru Hiramatsu, Shuichi Kaminogawa

**Affiliations:** 1 Technical Research Laboratory, Takanashi Milk Products Co., Ltd., Yokohama 241-0023, Japan; G-harata@takanashi-milk.co.jp (G.H.); Ke-miyazawa@takakanashi-milk.co.jp (K.M.); K-yoda@takanashi-milk.co.jp (K.Y.); M-hiramatsu@takanashi-milk.co.jp (M.H.); 2 Department of Food Bioscience and Biotechnology, College of Bioresource Sciences, Nihon University, Fujisawa 252-8510, Japan; ktaka@brs.nihon-u.ac.jp (K.T.); hosono@brs.nihon-u.ac.jp (A.H.); kamino@b-star.jp (S.K.)

**Keywords:** degranulation, IgE-mediated allergy, *Lactobacilli*, mast cell

## Abstract

Mast cells play a critical role in immunoglobulin E (IgE)-mediated allergic diseases, and the degranulation of mast cells is important in the pathogenesis of these diseases. A disturbance of the intestinal microflora, especially of endogenous lactic acid bacteria, might be a contributing factor for IgE-mediated allergic diseases. Additional knowledge regarding the interaction of human intestinal *Lactobacilli* with mast cells is still necessary. Twenty-three strains of *Lactobacilli*, including commercial and reference strains and strains from the human intestine, were tested for their ability to regulate degranulation of cells from rat basophilic leukemia RBL-2H3 cells (RBL-2H3) in vitro based on a β-hexosaminidase release assay. Each of the tested *Lactobacilli* characteristically suppressed IgE-mediated degranulation of RBL-2H3 cells, and *Lactobacillus* GG showed the strongest inhibitory effect on the cells. Furthermore, the bacteria isolated from the human intestine significantly suppressed degranulation of RBL-2H3 cellsin comparison with the reference strains. These results suggest that *Lactobacilli*, particularly those from the human intestine, can affect the activation of mast cells in a strain-dependent manner. Further study should be conducted to analyse the understanding mechanism.

## 1. Introduction

Allergic diseases are characterised by enhanced immunoglobulin E (IgE)-mediated responses to common environmental antigens. The prevalence of these diseases is increasing worldwide, particularly in western industrialised countries. The development of allergic disease is associated with lifestyle as well as environmental factors, and the increase of allergic diseases is paralleled to a decrease of exposure to microbial stimuli [1,2,3,4]. The evidence presented so far has implicated the intestinal microbiota in maintaining homeostasis and shaping the immune system, and dysbiosis of the intestinal microbiota is associated with the pathology of various allergic diseases and other autoimmune diseases [5,6,7]. Therefore, the intestinal microbiota is a possible therapeutic target for the management of allergic diseases.

Mast cells are multifunctional regulator cells that are located at normal connective tissue, blood vessels or nerves, or beneath epithelial surfaces, where these cells are exposed to the environment via the respiratory and gastrointestinal tracts. Mast cells comprise 2%–5% of mononuclear cells in the lamina propria of the normal gastrointestinal tract. Mast cells are well-known effectors of allergic responses, such as atopic dermatitis and asthma, and release many mediators such as histamine and produce pro-inflammatory cytokines [8]. High-affinity IgE receptor, also known as FcεRI, on mast cells plays a key role in the IgE-mediated type I hypersensitivity mediated by allergen cross-linking of the specific IgE-FcεRI complex. Thus, prevention of IgE binding to FcεRI on these cells is an effective therapy for allergic disease.Therefore, when developing anti-allergic pharmaceutical drugs, stabilisation of mast cells and suppression of degranulation should be the major targets. Recently, some food components from tea, fruit, seaweeds and vegetables have been proposed to alleviate symptoms of allergic diseases because these food components have the ability of inhibition for the activation of mast cells [9,10,11]. Furthermore, some of the non-pathogenic, commensal, intestinal mucosa-associated bacteria function as strong direct inhibitors of mast cell degranulation [12,13,14,15]. However, whether or not all the microorganisms have a suppressive effect remains unclear.

In this study, 23 strains of *Lactobacilli*, including commercial and reference strains and strains from the human intestine, were tested for their ability to suppress degranulation of mast cells using a cell line, rat basophilic leukemia RBL-2H3 cells (RBL-2H3), in vitro. Furthermore, the possible mechanisms by which these probiotic strains suppress the activation of mast cells were also explored.

## 2. Materials and Methods

### 2.1. Bacterial Preparation

The probiotic strain, *Lactobacillus rhamnosus* GG (LGG; ATCC 53103), was supplied by Valio Ltd. (Helsinki, Finland). *L. gasseri* TMC0356 (TMC0356) was isolated from the faeces of a healthy adult and stored at the Technical Research Laboratory of Takanashi Milk Products Co., Ltd. (Yokohama, Japan).

Nine reference strains of *Lactobacilli* were purchased from Japan Collection of Microorganisms (JCM), The Institute of Physical and Chemical Research (RIKEN; Wako, Japan) as shown in Table 1. Seven commercial strains of *Lactobacilli* were originally isolated from commercial fermented milk/yoghurt.

*Lactobacillus* sp. strains were previously isolated from five faecal samples obtained from the subjects who had been administered LGG- and TMC0356-fermented milk in clinical studies conducted in 2006 [16,17]. De Man-Rogosa-Sharpe (MRS) broth (Becton Dickinson, Sparks, MD, USA) was used to culture *Lactobacilli* at 37 °C for 18 h. After the incubation, cultured bacteria collected by centrifugation were washed three times with sterile saline, heat-killed at 100 °C for 30 min and lyophilised. Heat-killed bacteria were re-suspended in Eagle’s minimal essential medium (MEM; Wako, Japan) at a concentration necessary for each experiment.

### 2.2. Cell Culture

The RBL-2H3 cells(ATTC CRL-2256) were cultured in MEM supplemented with 10% heat-inactivated foetal bovine serum (FBS; Gibco, New York, NY, USA), 100 μg/mL streptomycin, 2 mM l-glutamine, 5 × 10^−5^ M 2-mercaptoethanol and 100 U/mL penicillin at 37 °C in a humidified incubator with 5% CO_2_.

### 2.3. Analysis of the β-Hexosaminidase Release

The release of β-hexosaminidase was measured for the degranulation of RBL-2H3 cells as described previously [18]. Briefly, the overnight cells (3.0 × 10^5^) with or without *Lactobacilli* (10 mg/mL) were cultured for 3 h at 37 °C and 5% CO_2_. The cells were washed with MEM and stimulated with monoclonal anti-2,4,6-trinitrophenyl (anti-TNP) IgE (clone IgE-3, 40 ng/mL; BD Pharmingen, Tokyo, Japan) for 2 h. the cells were washed with Tyrode’s buffer (126 mM NaCl, 5.6 mM glucose, 4.0 mM KCl, 0.6 mM KH_2_PO_4_, 10.0 mM 4-(2-hydroxyethyl)-1-piperazineethanesulfonic acid (HEPES), 0.6 mM MgCl_2_/6H_2_O, 1.0 mM CaCl_2_ and 0.1% bovine serum albumin (BSA)) and then stimulated with TNP-BSA (3 ng/mL; LSL, Tokyo, Japan) for 1 h at 37 °C. Culture supernatants were added to 0.2% Triton X-100 and incubated with 1.3 mg/mL *p*-nitrophenyl-*N*-acetyl-β-d-glucopyranoside (Nakarai Tesque, Kyoto, Japan) for 40 min at 37 °C. After developing the reaction with 0.2 M glycine, optical density at 450 nm was measured and the release of granules was calculated as the percentage of total β-hexosaminidase content determined using cell lysis with 0.2% Triton X-100. The release of β-hexosaminidase in the test sample was calculated using the following equation: Degranulation rate (%) = (T/A)/(C/A) × 100, where C (control) is antigen-induced β-hexosaminidase release in the absence of *Lactobacillus* [TNP-BSA(+) – TNP-BSA(−)], T (test) is the antigen-induced β-hexosaminidase of test sample [TNP-BSA(+) – TNP-BSA(−)], and A is total β-hexosaminidase content (Triton X-100 extract).

### 2.4. Binding of IgE

RBL-2H3 cells (5.0 × 10^5^) were seeded in a six-well plate and cultured overnight. The cells were washed with phosphate buffered saline (PBS), re-suspended in Dulbecco’s modified Eagle’s medium (DMEM) pH 7.2) containing 0.5% flow cytometry (FACS) BSA, 1 mM ethylenediaminetetraacetic acid (EDTA), 10 mM HEPES, 2 mM sodium pyruvate and 0.02% sodium azide and incubated with Alexa 488-labelled IgE on ice for 2 h in the presence of various amounts of *Lactobacilli* (0.01, 0.1 or 1.0 mg/mL). Mouse IgE was labelled with Alexa 488 using a labelling kit purchased from Invitrogen (Carlsbad, CA, USA). The cells were stained with Alexa 488-labelled IgE were analysed using flow cytometry.

### 2.5. Cytokine Production

RBL-2H3 cells (3.0 × 10^5^) were seeded in a 24-well plate and incubated overnight. After treatment with various amounts of *Lactobacilli* (0.01–1.0 mg/mL) for 3 h and with IgE (0.2 µg/mL) for 2 h, the cells were washed twice with MEM and stimulated with TNP-BSA antigen (30 ng/mL) for 3 h (for TNF-α release) or 6 h (for IL-13 release). Concentrations of TNF-α and IL-13 in the culture supernatants were determined using ELISA kits (BioSource; Camarillo, CA, USA) according to the manufacturer’s instructions.

### 2.6. Western Blotting

RBL-2H3 cells (5.0 × 10^5^) were seeded in a six-well plate and cultured overnight. After treatment with or without *Lactobacilli* (1.0 mg/mL) for 3 h, the cells were washed with MEM and stimulated with IgE (0.2 µg/mL) for 2 h at 37 °C. Next, the cells were washed with MEM and stimulated with TNP-BSA antigen (3 ng/mL) for 5 min at 37 °C. The cells were then washed with ice-cold PBS and incubated on ice for 10 min in the lysis buffer (20 mM Tris, pH 7.6, 1% Nonidet P-40, 60 mM octyl-B-glucoside, 50 mM NaF, 1 mM sodium orthovanadate, 2 mM phenylmethylsulphonyl fluoride, 10 mg/mL aprotinin, 2 mg/mL leupeptin and 2 mg/mL pepstatin). Proteins were separated by sodium dodecyl sulphate-polyacrylamide gel electrophoresis (SDS-PAGE) and transferred to polyvinylidene difluoride membranes for immunoblotting. A primary antibody was used as phosphotyrosine. A horseradish peroxidase-conjugated anti-rabbit IgG antibody served as a secondary antibody. The antibodies were purchased from Cell Signaling Technology (Beverly, MA, USA).

### 2.7. Statistical Analysis

The statistical significance of the differences between the two groups was calculated using unpaired Student’s *t*-test or Welch’s *t*-test after an *F*-test.

## 3. Results

### 3.1. Inhibition of IgE-Mediated Degranulation by Lactobacilli

**Figure 1:**
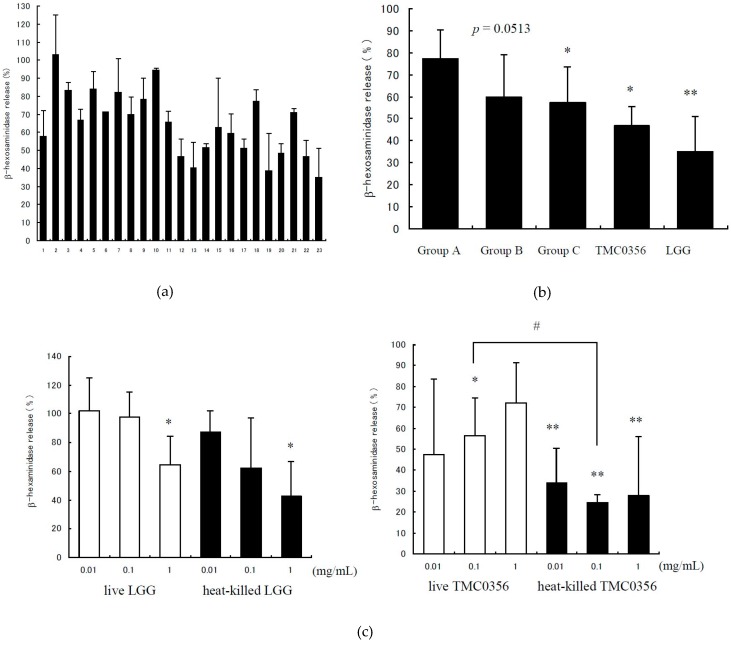
Degranulation rates of each *Lactobacillus* according to β-hexosaminidase release. RBL-2H3 cells were pre-incubated with each *Lactobacillus* strain for 3 h, prior to IgE sensitisation. After stimulation with the antigen, degranulation was detected by measuring the release of β-hexosaminidase. The control is antigen-induced β-hexosaminidase release in the absence of *Lactobacillus*, calculated as 100%. (**a**) Strain No. 22 is LGG, and 23 is TMC0356. Group A (strain No. 1–9) consists of the reference strains purchased from JCM, Group B (strain No. 10–16) consists of commercial strains isolated from fermented milk/yoghurt and Group C (strain No. 17–21) consists of *Lactobacilli* obtained from the human intestine. (**b**) Comparison of degranulation rates among treatment groups: TMC0356 and LGG. (**c**) Degranulation rates (dose-response relationship) of live or heat-killed LGG and TMC0356 (0.01, 0.1 or 1.0 mg/mL). The results are presented as mean ± standard deviation (SD) (*n* = 3). **, *p* < 0.01, *, *p* < 0.05 for comparison of the control without *Lactobacillus* treatment; #, *p* < 0.05 for comparison to live LGG or TMC0356.

The degranulation of RBL-2H3 cells was detected by measuring the release of β-hexosaminidase.The positive control showed 8.94% ± 2.36% (mean ± standard deviation (SD)) in this study and was calculated as 100%. Each of the 23 tested *Lactobacilli* indicated that IgE-mediated degranulation of the lysate of the RBL-2H3 cells was released by 35.0%–102.9%, with each strain showing its own characteristic inhibitory effect (Figure 1a). Of the tested *Lactobacilli*, LGG (strain No. 23) showed the strongest inhibitory effect on the degranulation of RBL-2H3 cells, whereas *L. reuteri* JCM 1112 (strain No. 2) did not inhibit the degranulation of RBL-2H3 cells. Group C contained the *Lactobacilli* originally isolated from the human intestine; these bacteria significantly suppressed IgE-mediated degranulation in comparison with the reference strains in Group A (*p* < 0.05). Furthermore, Group B which contained the commercial strains also tended to suppress the degranulation in comparison with the reference strains in Group A (*p* = 0.0513) (Figure 1b).

The inhibitory effects of the live and heat-killed strains LGG on IgE-mediated degranulation of RBL-2H3 cells were dose-dependent; the heat-killed TMC0356 showed significant inhibition atthe dosage of 0.01, 0.1 and 1.0 mg/mL (Figure 1c). There were significant differences between the effects of heat-killed and live TMC0356 at the dosage of 0.1 mg/mL. Possible toxic effects or damage of the bacteria preparation to tested RBL-2H3 cells were not observed.

### 3.2. Effect of LGG and TMC0356 on Binding of IgE to the Surface of RBL-2H3 Cells

**Figure 2:**
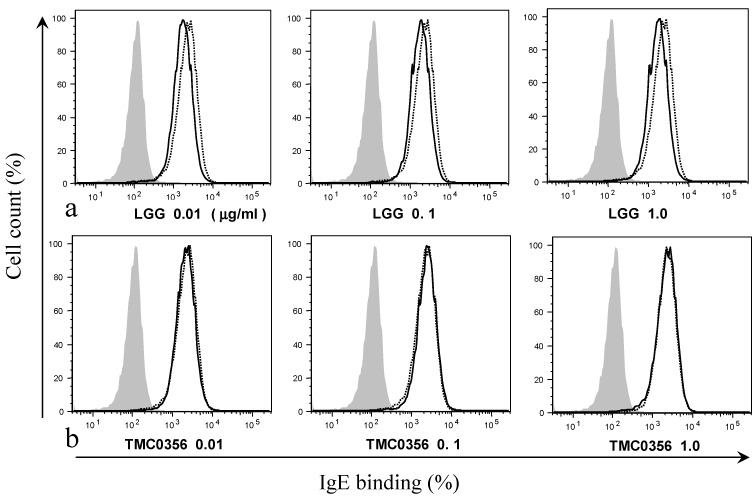
Effect of LGG and TMC0356 on binding of IgE to the surface of RBL-2H3 cells.RBL-2H3 cells were analysed using flow cytometry to evaluate the influence of LGG or TMC0356 (0.01, 0.1 or 1.0 mg/mL). Shadowed areas = no IgE; dotted lines = with IgE but without LGG and TMC0356; bold lines = with IgE and either LGG (**a**) or TMC0356 (**b**). The results of one of three independent experiments with similar results are shown.

To investigate whether the suppression of IgE binding could account for the LGG- and TMC0356-induced inhibition of degranulation, RBL-2H3 cells were incubated with LGG or TMC0356 and sensitised using IgE. The treatment with LGG slightly decreased the binding of IgE to the surface of RBL-2H3 cells, whereas the cells exposed to TMC0356 showed no significant decrease in IgE binding regardless of the dose (Figure 2).

### 3.3. Effect of LGG and TMC0356 on Cytokine Production

The release of TNF-α from RBL-2H3 cells after antigen stimulation was slightly inhibited by pre-treatment with TMC0356 and LGG. However, the difference was not significant (Notreatment 28.93 ± 7.55 pg/mL; LGG, 1.0 mg/mL, 17.64 ± 13.57 pg/mL; TMC0356, 1.0 mg/mL, 11.13 ± 7.85 pg/mL). Furthermore, neither LGG nor TMC0356 inhibited the release of IL-13 (No treatment 560.06 ± 406.18 pg/mL; LGG 1.0 mg/mL treatment 476.62 ± 236.53 pg/mL; TMC0356 1.0 mg/mL treatment 417.92 ± 146.56 pg/mL).

### 3.4. Effect of LGG and TMC0356 on Intracellular Signalling

The signalling cascade associated with the receptor is initiated when the immunoreceptor tyrosine-based activation motif of the β and γ chains are phosphorylated on a tyrosine. This signal is required for the activation of mast cells; therefore, tyrosyl transphosphorylation, which occurs during the early stages of signal transduction, is considered an important marker of mast cell activation.

**Figure 3:**
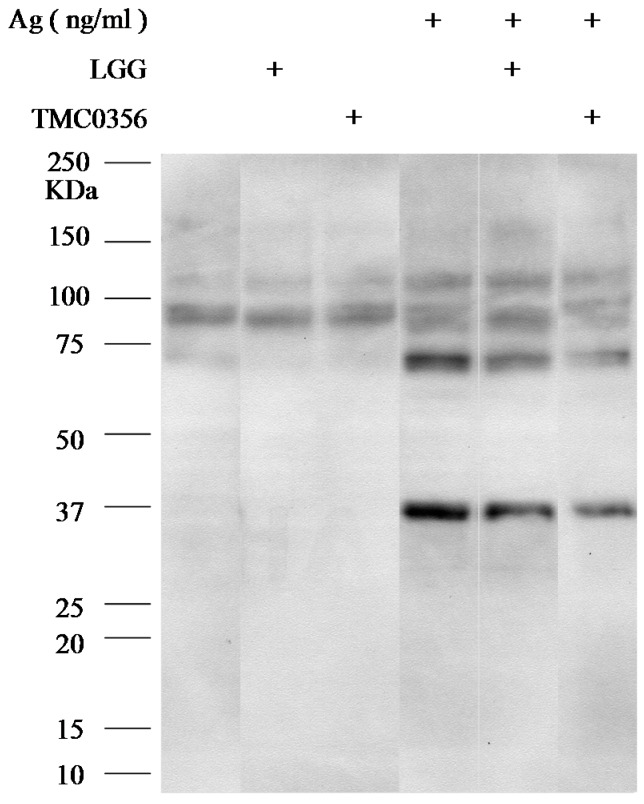
Effect of LGG and TMC0356 on intracellular signalling.RBL-2H3 cells were incubated with heat-killed LGG or TMC0356 (1.0 mg/mL). After the cells were sensitised with IgE and stimulated with the antigen for 5 min, cell lysates were prepared and immunoblotted with anti-phosphotyrosine antibodies. The results of one of three independent experiments with similar results are shown.

To investigate the mechanisms underlying the inhibition of degranulation of RBL-2H3 cells by LGG and TMC0356, RBL-2H3 cells were incubated with LGG or TMC0356, sensitised with IgE and the antigen, and immunoblotted with anti-phosphotyrosine antibodies to determine the molecular mechanisms by which LGG and TMC0356 inhibit mast cell activation. Total tyrosine phosphorylation patterns were not significantly affected by LGG or TMC0356 treatment (Figure 3).

## 4. Discussion

LGG is a probiotic strain with well-documented anti-allergic effects. This bacterium effectively protects infants with a genetically high risk of allergic diseases caused by the development of atopic diseases [19,20,21], and it also alleviates atopic eczema-dermatitis syndrome by enhancing interferon-γ responses of peripheral lymphocytes in infants with cow milk allergy or IgE-associated atopic eczema-dermatitis syndrome [22,23]. However, LGG does not significantly affect birch pollen allergy, an adult allergic disease [24]. TMC0356 was originally isolated from the intestine of a healthy adult [25]; these bacteria generally adhere to human enterocytes and do not enhance inflammatory responses [26]. TMC0356 characteristically induces the secretion of pro-inflammatory (IL-12) and anti-inflammatory (IL-10) cytokines by murine macrophages [27]. This bacterium effectively inhibited antigen-augmented serum IgE in BALB/c mice that was immunised intra-peritoneally with the food antigen, ovalbumin, and it altered the serum IgE concentration of the subjects with high serum IgE levels and perennial allergic rhinitis [28,29].

LGG-TMC0356-fermented milk significantly suppressed ovalbumin (OVA)-induced nasal vascular permeability and the non-specific IgE level in rats [30], and alleviated OVA-induced nasal blockage in guinea pigs [31]. In 2006, the LGG- andTMC0356-fermented milk was orally administered to patients with Japanese cedar pollinosis (JCPsis) in a double-blind, placebo-controlled clinical trial during the season of Japanese cedar pollen. Some clinical improvements were observed after this treatment. However, no significant changes in the levels of serum IgE and other blood biomarkers related to IgE immunity were observed in this clinical study, although LGG and TMC0356 suppressed IL-4 and IL-5 production by peripheral blood mononuclear cells isolated from patients with JCPsis when the cells were stimulated with both CryJ1 and PHA in vitro [17].

On the other hand, oral administration of LGG- and TMC0356-fermented milk suppressed changes in intestinal microbiota in patients with JCPsis in the same clinical study [16]. Therefore, the oral administration of LGG and TMC0356 presumably resulted in intestinal colonisation by these bacteria in all patients with JCPsis that were administered the LGG- and TMC0356-fermented milk [17]. These studies indicate that the intestinal microbes that are protected by LGG and TMC0356, which had colonised the intestinal tract of the patients with JCPsis, may be involved in the alleviation of the clinical symptoms. However, the analysis of blood conducted in the study was not sufficient to confirm that notion. Therefore, additional information regarding the effects of LGG, TMC0356 and intestinal microbes in patients with JCPsis on immune cells is required to understand the mechanism underlying the anti-allergic effect of LGG and TMC0356.

Recently, human intestinal bifidobacteria showed a characteristic inhibitory effect against active mast cells using RBL-2H3 cells. Compared to the infant-specific species, *Bifidobacterium bifidum*, which strongly inhibits degranulation, the adult-specific species, *B. adolescentis*, showed variation in its ability to affect IgE-mediated degranulation among different strains [18]. These results indicate that RBH-2H3 cells are highly sensitive to different properties of intestinal microbes to implicate the activation of mast cells.

In the present study, each strain of the tested *Lactobacilli* characteristically suppressed IgE-mediated degranulation of RBL-2H3 cells. Among the tested bacteria, LGG and TMC0356 showed a stronger inhibitory effect on the degranulation of RBL-2H3 cells than that of other strains. These inhibitory effects were dose-dependent and not associated with the viability of these bacteria. The tested bacteria that were originally isolated from the human intestine, including those isolated from the patients with JCPsis, significantly suppressed degranulation of RBL-2H3 cells compared with other bacteria. These results indicate that *Lactobacilli*, particularly some of the selected strains, may possess the ability to alter activation of mast cells. Orally administered LGG and TMC0356 can successfully colonise the intestine of patients with JCPsis and stabilise other beneficial intestinal microbes, including *Lactobacilli* and bifidobacteria. The inhibition of degranulation of mast cells may be a part of the underlying mechanism by which these microbes alleviate the clinical symptoms of patients with JCPsis, as reported in the previous study. These results are also supported by another study [15] showing that probiotics exert potential anti-allergic effects, at least in part, through direct action on mast cells.

To explore the mechanisms underlying the inhibition of degranulation of RBL-2H3 cells by LGG and TMC0356, LGG and TMC0356 were tested for the ability to affect the tyrosine phosphorylation, TNF-α production and binding of IgE to RBL-2H3 cells. TMC0356 tended to suppress the secretion of TNF-α, whereas LGG slightly inhibited the interaction between IgE and FcεRI. However, these changes were not significant and therefore could not sufficiently explain the mechanism underlying the inhibitory effects of LGG and TMC0356.LGG can significantly downregulate the expression of the genes of high-affinity IgE receptor subtype α (FCER1A) and HRH14, affecting the function of human mast cells, as observed previously by microarray analysis [32]. A Toll-like receptor 2 (TLR2) ligand significantly suppresses the FcɛRI-mediated inflammatory responses of mast cells [15]. Further studies will be conducted to test whether LGG and TMC0356 can alter the activation of mast cells by suppressing expression of the *FCER1A* and *HRH14* genes or by interacting with TLR2.

## 5. Conclusions

The results obtained from the present study suggest that *Lactobacilli*, particularly those from the human intestine, affect the activation of mast cells in a strain-dependent manner and by different mechanisms to express the anti-allergic effects.

## Figures and Tables

**Table 1:** The tested strains of *Lactobacilli* is olated from the human intestine, commercial and reference strains.

No.	Microorganism
1	*Lactobacillus brevis* JCM1059 ^T^
2	*Lactobacillus reuteri* JCM1112 ^T^
3	*Lactobacillus gasseri* JCM1131 ^T^
4	*Lactobacillus acidophilus* JCM1132 ^T^
5	*Lactobacillus casei* subsp. *Casei* JCM1134 ^T^
6	*Lactobacillus casei* subsp. *Rhamnosus* JCM1136 ^T^
7	*Lactobacillus plantarum* JCM1149 ^T^
8	*Lactobacillus salivarius* subsp. *Salivarius* JCM1231 ^T^
9	*Lactobacillus johnsonii* JCM2012 ^T^
10–16	*Lactobacilli* isolated from fermented milk/yoghurt
17–21	*Lactobacilli* isolated from human fecal
22	*Lactobacillus gasseri* TMC0356
23	*Lactobacillus rhamnosus* GG

T: Type strain.
